# Genus *Curcuma*: chemical and ethnopharmacological role in aging process

**DOI:** 10.1186/s12906-023-04317-w

**Published:** 2024-01-11

**Authors:** Esraa A. Elhawary, Ashaimaa Y. Moussa, Abdel Nasser B. Singab

**Affiliations:** 1 Department of Pharmacognosy, Faculty of Pharmacy, Ain Shams University, Cairo, 11566 Egypt; 2 Center for Drug Discovery Research and Development, Ain Shams University, Cairo, 11566 Egypt

**Keywords:** *Curcuma*, Zingiberaceae, Anti-aging, Ethnobotany, Neuroprotective

## Abstract

Aging or senescence is part of human life development with many effects on the physical, mental, and physiological aspects which may lead to age-related deterioration in many organs. Genus *Curcuma* family Zingieraceae represents one of the well-studied and medically important genera with more than eighty species. The genus is reported to contain different classes of biologically active compounds that are mainly presented in diphenylheptanoids, diphenylpentanoids, diphenylalkanoids, phenylpropene derivatives, alkaloids, flavonoids, chromones, terpenoids, phenolic acids and volatile constituents. Rhizomes and roots of such species are rich with main phytoconstituents *viz.* curcumin, demethoxycurcumin and bis-demethoxycurcumin. A wide variety of biological activities were demonstrated for different extracts and essential oils of genus *Curcuma* members including antioxidant, anti-inflammatory, cytotoxic and neuroprotective. Thus, making them as an excellent safe source for nutraceutical products and as a continuous promising area of research on lead compounds that may help in the slowing down of the aging process especially the neurologic and mental deterioration that are usually experienced upon aging. In this review different species of the genus *Curcuma* were summarized with their phytochemical and biological activities highlighting their role as antiaging agents. The data were collected from different search engines *viz.* Pubmed®, Google Scholar®, Scopus® and Web of Science® limiting the search to the period between 2003 up till now.

## Introduction

Medicinal plants were utilized for decades as medicine and curative agents for human beings. Aging is the sequential or progressive change in an organism with continuous process of natural change usually begins in early adulthood which leads to an increased risk of debility, disease, and death. Senescence consists of a lot of manifestations related to the aging process. Aging is an inevitable process that includes a progressive and irreversible decrease in physiological abilities which leads to many diseases such as cardiovascular diseases, musculoskeletal disorders and arthritis, neurodegenerative diseases, and cancer. Different mechanisms had postulated the role of aging in cancer development including oxidative stress, DNA damage and mutation, cell aging, decreased immunity, one or all of them may lead to cancer development by aging [1, 2].

**Fig. 1:**
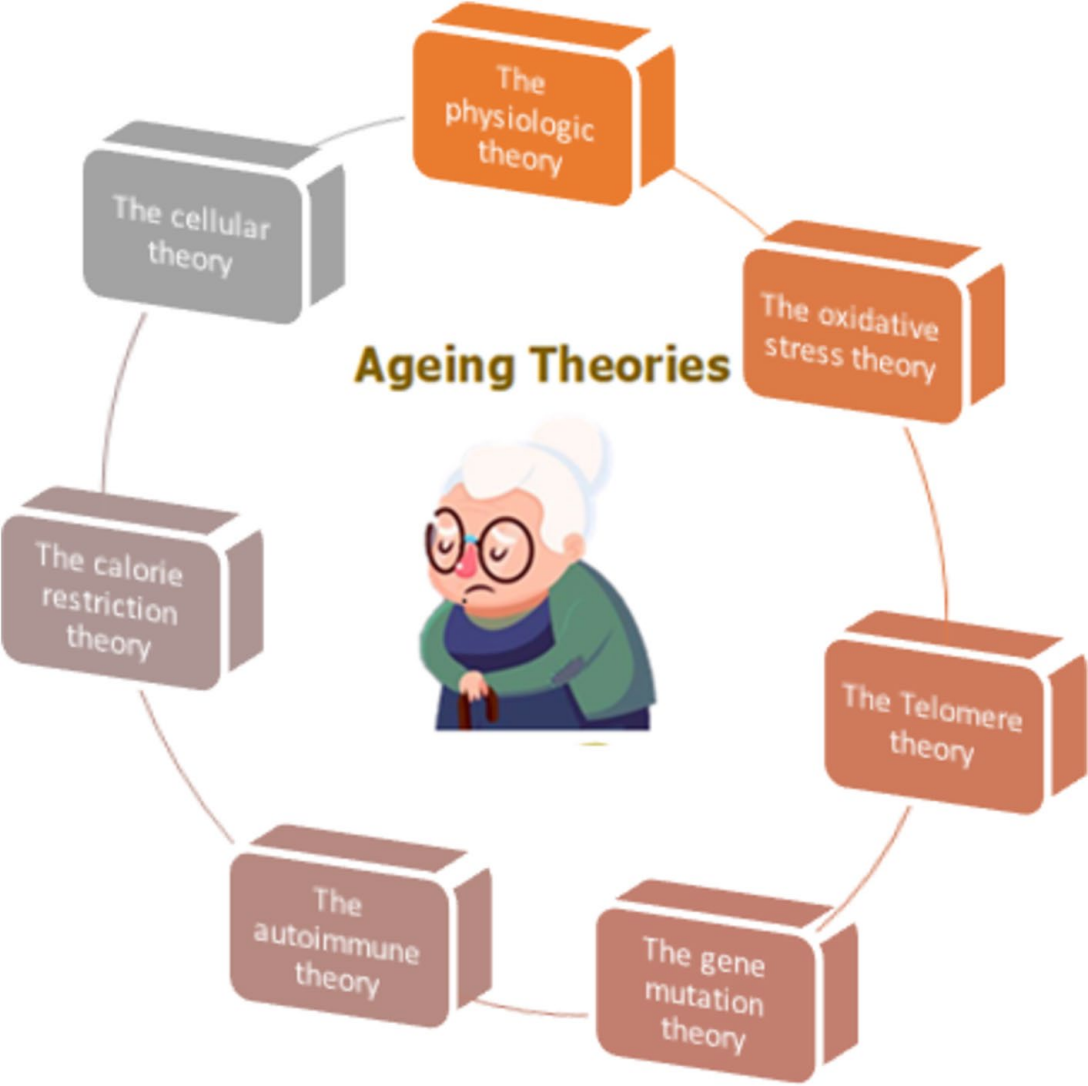
Diagram showing different theories of the aging process

Several theories were exploited in order to understand the aging sequences and consequences. Aging can be attributed to one of such theories or a punch of them (Fig. 1) [3].

1-The cellular theory (Fig. 1) includes intrinsic timing mechanisms and signals, accidental events, programmed genetic signals, damage in DNA or protein structure, waste accumulation or general molecular wear and tear. 2-The physiologic theory (Fig. 1) includes oxidative stress, immunologic, neuro endocrinologic, metabolic, and insulin signaling, and caloric restriction [3].

3-The oxidative stress theory (Fig. 1) is the most common explanation over the last decade evaluating the role of the antioxidant vitamins such as B_12_, folic acid, A, C, D and E and their effect in slowing oxidative stress [4–6]. This theory was emphasized through reversal of the oxidative stress pathway, which may lead to extended life span [7].

4-The gene mutation theory (Fig. 1) explains that deletions, mutations, translocations, and polyploidy are aged-related chromosomal abnormalities that may end in either gene slowing or expression of specific genes that activate certain types of cancers [7].

5-The autoimmune theory (Fig. 1) involved the idea that the human body started to produce autoantibodies to its own tissues and/or deficit primarily in T-cell function led to the development of infections, chronic disease, and cancer, especially autoimmune diseases such as rheumatoid arthritis and systemic *lupus erythematosus* [8].

6-The calorie restriction theory (Fig. 1) and mutations in insulin-signaling pathways might result in alterations in body size and composition, enhanced resistance to oxidative stress. 7- Telomere theory (Fig. 1) stated that the telomerase enzyme added telomere repeats to the ends of chromosomes. Critical shortening of the telomeric DNA owing to loss of the enzyme telomerase was the signal for the initiation of cellular senescence [9]. Despite the many theories trying to explain the aging process a lot of mechanisms are still not understood and the aging process usually differ for the same person and from one person to another [10].

A lot of research and review articles has been published with the terms “aging” or “antiaging” in order to either explain the aging process or try to find a solution to the process flow or outcomes. Moreover, many natural compounds and extracts carried a potential antiaging activity through affecting the oxidative stress, inflammation, neurologic function, cardiovascular system, *etc*. The antiaging activity of such natural agents can be proved through many mechanisms, in vitro and in vivo models [11, 12].

Plants that act as antiaging carry a lot of valuable active ingredients that may help reverse the aging process. Polyphenolic compounds represent an important class of phytoconstituents and they possess potent biological activities like antioxidant, anti-inflammatory and anticancer properties which play a role in delaying, preventing or treating aging-related diseases such as cancers, nephrosclerosis, arthritis, neurodegenerative and cardiovascular diseases [13]. The main antiaging polyphenolics include quercetin, luteolin, catechins, curcumin, resveratrol and lignans. Moreover, other classes of phytoconstituents were reported as antiaging agents such as saponins from *Panax ginseng*, *Gynostemma pentaphyllum* and *Glycyrrhiza glabra*, and in few marine organisms such as starfish and sea cucumbers in addition to alkaloids, polysaccharides and essential oils [14].

Macro- and micronutrients such as vitamins, minerals (as micronutrients), essential and branched amino acids, polyunsaturated fatty acids (PUFA), probiotics together with the above mentioned phytoconstituents are well-known sources of antiaging natural sources. They provide this *via* reversal of oxidative stress in the body as well as their effects on the atherosclerosis caused by chronic inflammation thus preventing or delaying age-related diseases such as diabetes, cancer, neurodegeneration, and cardiovascular illnesses [15]. Genus *Curcuma* is rich in many documented antioxidants and anti-inflammatory active constituents with reported antiaging activities such as curcumin which has a key role played in aging reversal through its antioxidant, immunomodulatory and anti-inflammatory properties [10, 16]. Moreover, the promising neuroprotective activity of curcumin was reported against many neurodegenerative diseases *viz.* Alzheimer’s, Parkinson’s and multiple sclerosis [17]. Thus, genus *Curcuma* was chosen here in this review article in order to shed light on its main active phytoconstituents with particular use as antiaging natural source. This review article for the first time addresses the relation between the biological activity of the genus *Curcuma* in relation to aging with emphasis on the main active ingredients responsible for this activity.

### Ethnopharmacology

Genus *Curcuma* is one of the most popular and important genera of the family Zingiberaceae, which is comprised of 93 species. Genus *Curcuma* L. is composed of perennial and rhizomatous species. It is naturally found in the tropical and subtropical regions and is mainly distributed in India, Thailand, China, Malaysia, Indonesia and Northern Australia. Genus *Curcuma* members carry unique inflorescence with compound spike and prominent bracts each subtending a cincinnus of two-ten flowers, which are attached to each other forming pouches at the base. Several *Curcuma* species are famous and familiar with their medicinal uses including; *C. longa, C. zedoaria, C. amada*, *C. aromatic*, *C. angustifolia, C. neilgherrensis, C. kudagensis, C. thalakaveriensis, C. pseudomontana* and *C. coriacea*, *C. aeruginosa, C. angustifolia, C. amada, C. aromatic, C. australasica*, *C. caesia, C. mangga* and *C. sumatrana* [18, 19].

*Curcuma* contains many phytoconstituents namely, phenolics, alkaloids, diaryl pentanoids and essential oils. *Curcuma* has been widely used in folk medicine, especially in the Islamic era, for the treatment of many skin and liver conditions also for hemorrhoids, asthma, inflammation and leprosy. Curcumin is a natural polyphenolic compound isolated from genus *Curcuma* with different biological and pharmacological properties including antioxidant, immunostimulant, anti-inflammatory, anti-microbial, cardiovascular, renal and liver-protective, anti-cancer, anti-rheumatic, and anti-aging [10].

A lot of species belonging to the genus *Curcuma* were reported to carry interesting folk medicinal uses and some of them can be summarized as follows. *Curcuma longa* (Turmeric), is commonly used in folk medicine for many ailments *viz.* cancer, diabetes, arthritis, diarrhoea, inflammation, psoriasis, hepatobiliary diseases, sore throats, rheumatism, antiseptic, gastric and peptic ulcers which are usually related to many valuable phytoconstituents mainly curcumin [20]. Turmeric has been widely used in South and Southeast Asia for thousands of years. It has been valued as a spice in ‘curry’ especially in marriage and religious ceremonies for Indian people. Nowadays, turmeric is much valued for healthy beverages such as turmeric latte, aka and haldi doodh [21–24]. *Curcuma caesia* also known as black turmeric carried a lot of uses in folk medicine for many conditions such as asthma, fever, cancer, wounds, allergies, toothache, leprosy, bronchitis, epilepsy, hemorrhoids, leukoderma and rheumatoid arthritis. *C. caesia* has been found to be medicinally active as anti-bacterial in acne, analgesic, worm repellent, anti-asthmatic, anti-inflammatory, anti-bacterial, anti-fungal, anti-diabetic, anti-proliferative, anticancer, anti-ulcer, hepatoprotective, nephroprotective, neuroprotective and cytotoxic agent [25]. *Curcuma aeruginosa* Roxb. rhizome has been reported in traditional medicine as a disinfectant, expectorant, and tonic. Moreover, it has been used for wound healing, diarrhea, dysmenorrhea, fever, coughs, and asthma. Researchers have reported the medicinal use of this rhizome as anticancer, antioxidant, antimicrobial, anti-dengue, immunostimulant, anthelmintic, anti-inflammatory, anti-androgenic, analgesic, uterine relaxant and antipyretic [26].

*Curcuma zedoaroides* rhizomes were traditionally used by Thai people as antiseptic and wound healing aid against snake bites by king Cobra. The inhibitory effect of *C. zedoaroides* extract and its fractions on inflammation were detected by reduction of nitric oxide release using RAW264.7 cells. The improvement capabilities on wound healing were determined on fibroblast L929 cells proliferation and migration assays (IC_50_ = 14.0–14.6μg/ml) [27]. *Curcuma zedoaria* Rosc is a perennial herb found in tropical countries, such as India, Japan and Thailand. Different organs of this species are utilized in Ayurveda and other folk medicines for the treatment of different ailments such as diarrhoea, cancer, flatulence and dyspepsia [28]. *Curcuma angustifolia* Roxb. (Indian Arrowroot) is widely distributed in India and some parts of Nepal, Thailand, Bangladesh and Pakistan. It is traditionally used as medicine for treating various diseases and also used as food. Few data are available about its application in pharmacology and therapeutics. Secondary metabolites found in Indian Arrowroot include essential oils, alkaloids, flavonoids, terpenoids, phytosterols, terpenes, phenols, and others. Pharmacological activities such as antioxidant, anti-inflammatory, anti-proliferative, anti-ulcerogenic, hepatoprotective, and anti-cancerous activities have been shown [29].

### Recent advances in the field of antiaging natural products

Anti-aging research projects are usually targeting certain age-related disorders such as Parkinson's Disease (PD), Alzheimer's Disease (AD), cardiovascular diseases, cancer, and chronic obstructive pulmonary diseases. The causes of such disorders and illnesses are numerous and many molecular mechanisms are involved such as telomere shortening, NF-κB pathway, adiponectin receptor pathway, insulin, and IGF signaling pathway, AMPK, *m*TOR and mitochondrial dysfunction [30].

Recently, three signaling pathways that can help delay aging were discovered including the lower activity of insulin/IGF-1 (insulin-like growth factor) signaling, mTOR (mammalian target of rapamycin) nutrient sensing signaling networks and “NAD World” (NAD, nicotinamide adenine dinucleotide) or NAMPT/NAD/SIRT1, an ubiquitous network involved in the metabolic homeostasis of aging consisting of two parts: NAD +—dependent protein deacetylases SIRT1 (sirtuin) and NAMPT (nicotinamide phosphor-ribosyl-transferase), a driver that mediated NAD biosynthesis. mTOR integrates at least two functionally different complexes in mammals, in particular mTORC_1_ (mTOR complex 1) and mTORC_2_ (mTOR complex 2) [31].

Different marine sources acted as a new and effective means of antiaging natural products isolation. Crustaceans and seaweeds can provide potent antioxidants such as carotenoids and phenolic compounds. The carotenoid, astaxanthin, isolated from crustaceans or other marine organisms had potential antioxidant and anti-wrinkle activity against skin aging *via* enhancing skin elasticity and reducing wrinkle formation due to immune-modulating, anti-inflammatory and DNA repair effects. Astaxanthin can also prevent neurodegenerative disorders. Laminarin extracted from brown algae attenuates UV-induced skin damage. In addition to that, marine microorganisms such as microalgae, bacteria and myxomycetes can be sources of antibacterial, antiviral, antitumoral and antioxidant phytoconstituents. The green microalgae named *Dunaliella salina* can delay skin aging owing to its anti-inflammatory and antiglycation effects while its carbohydrate content can benefit skin health [15].

## Role of genus *Curcuma* members in aging

### Phytoconstituents

#### Diphenylheptanoids (Diarylheptanoids) (Fig. 2)

**Fig. 2:**
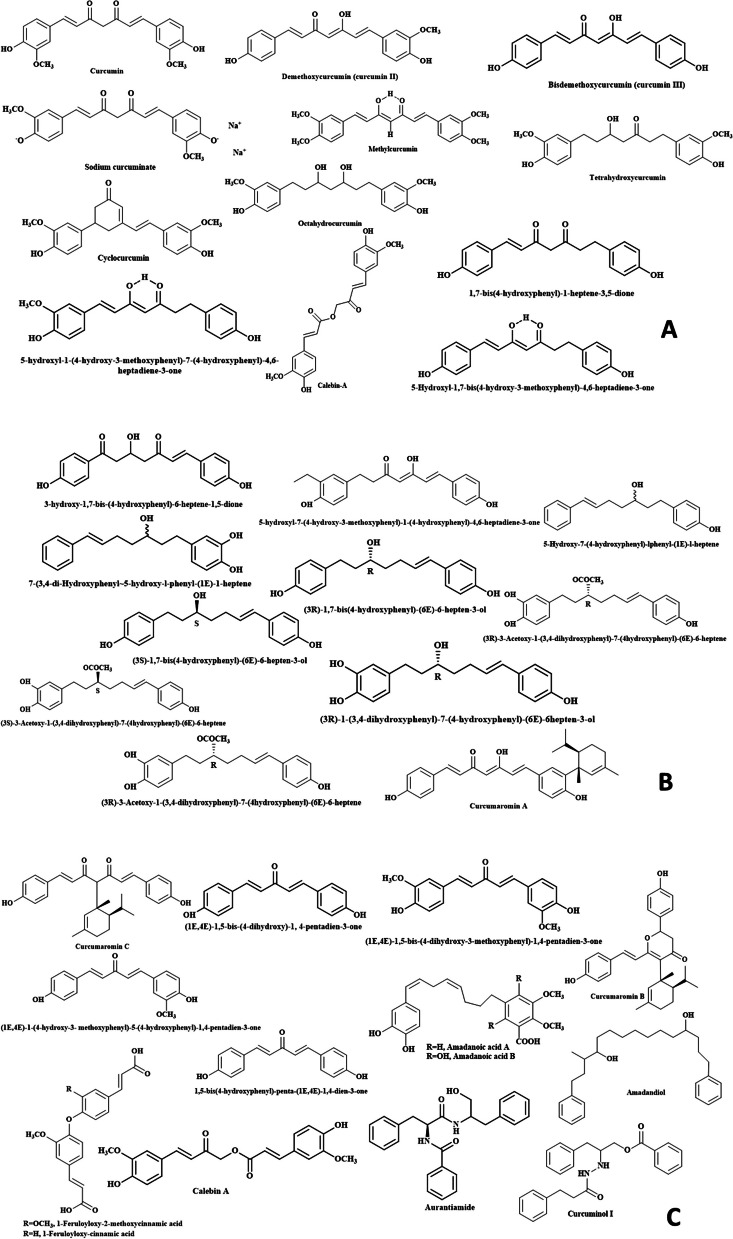
Summary of the main identified phytoconstituents from different species of genus *Curcuma*

Diphenylheptanoids (diarylheptanoids) represents one of the main identified chemical classes from genus *Curcuma.* This class is best represented by curcumin (curcumin I) which was reported to be isolated from the rhizomes of *C. aromatica* (hydromethanol extract of the rhizome through reversed phase HPLC, ethyl acetate extract of the rhizome)*, C. longa* (methanol extract)*, C. manga* (methanol extract, hexane and ethyl acetate fractions)*, C. xanchorrhiza* (essential oil extracted by supercritical CO_2_) and *C. zedoaria* (ethyl acetate fraction) [24, 32–39]. Many different derivatives belonging to the diarylheptanoid class were isolated from different *Curcuma* species and can be detailed herein. Most of the diarylheptanoids were reported from *C. longa* rhizome *viz.* demethoxycurcumin (curcumin II) [24, 33, 35, 37, 40, 41], bisdemethoxycurcumin (curcumin III) [42] [24, 37, 38, 41], methylcurcumin, sodium curcuminate [35], octahydrocurcumin (*C. longa,* leaves) [43], tetrahydroxycurcumin [44], cyclocurcumin, calebin-A, 1-(4-hydroxy-3-methoxyphenyl)-7-(3, 4-dihydroxyphenyl)-1,6-heptadiene-3, 5-dione and 1-(4-hydroxyphenyl)-7-(3, 4-dihydroxyphenyl)-1,6-heptadiene-3, 5-dione [24, 38, 45], 5-hydroxyl-1-(4-hydroxy-3-methoxyphenyl)-7-(4-hydroxyphenyl)-4,6-heptadiene-3-one, 5-hydroxyl-1,7-bis(4-hydroxy-3-methoxyphenyl)-4,6-heptadiene-3-one, 1,7-bis-(4-hydroxyphenyl)-1-heptene-3,5-dione, 3-hydroxy-1,7-bis-(4-hydroxyphenyl)-6-heptene-1,5-dione and 5-hydroxyl-7-(4-hydroxy-3-methoxyphenyl)-1-(4-hydroxyphenyl)-4,6-heptadiene-3-one [46]. In addition to that, 5-hydroxy-7-(4-hydroxyphenyl)-lphenyl-(1*E*)-l-heptene and 7-(3,4-dihydroxyphenyl ~ 5-hydroxy-l-phenyl-(1*E*)-1-heptene were isolated from *C. xanchorrhiza* (rhizome) [38] while (3*S*)-1,7-bis-(4-hydroxyphenyl)-(6*E*)-6-hepten-3-ol, (3*R*)-1,7-bis(4-hydroxyphenyl)-(6*E*)-6-hepten-3-ol, (3*S*)-1-(3,4-dihydroxyphenyl)-7-(4-hydroxyphenyl)-(6E)-6hepten-3-ol, (3*R*)-1-(3,4-dihydroxyphenyl)-7-(4-hydroxyphenyl)-(6E)-6hepten-3-ol, (3*S*)-3-acetoxy-1-(3,4-dihydroxyphenyl)-7-(4hydroxyphenyl)-(6E)-6-heptene and (3*R*)-3-acetoxy-1-(3,4-dihydroxyphenyl)-7-(4hydroxyphenyl)-(6E)-6-heptene were identified from the rhizome of *C. kwangsiensis* [47]. Moreover, curcumaromin A, curcumaromin B and curcumaromin C were reported from *C. aromatica* rhizome [35] but demethoxycurcumin was defined the rhizomes of *C. aromatica* rhizome, *C. amada* and *C. manga* [24, 33, 35, 37, 40, 41]. From *C. aeruginosa* rhizome, tetrahydro-bisdemethoxycurcumin was identified [48]. Octahydrocurcumin was also isolated from *C. mangga* (leaves) [43] while bis-demethoxy curcumin from *C. amada* and *C. manga* rhizomes [24, 37, 38, 41].

#### Diphenylpentanoids (Fig. 2)

Most of the diphenylpentanoids isolated from genus *Curcuma* were from *C. longa.* Three diphenylpentanoids were isolated and identified from the rhizome methanol extract of *C. longa* namely; (1E,4E)-1,5-bis-(4-dihydroxy)-1, 4-pentadien-3-one, (1E,4E)-1,5-bis-(4-dihydroxy-3-methoxyphenyl)-1,4-pentadien-3-one and (1E,4E)-1-(4-hydroxy-3-methoxyphenyl)-5-(4-hydroxyphenyl)-1,4-pentadien-3-one [49–54]. In addition to that, 1,5-bis-(4-hydroxyphenyl)-penta-(1E,4E)-1,4-dien-3-one was reported from the root ethyl acetate extract of the same species [55].

#### Diphenylalkanoids (Fig. 2)

The extract of *C. amada* rhizome yielded three diphenylalkanoids which were named following the species name as amadanoic acid A (aryl-C9-aryl skeleton), amadanoic acid B (aryl-C9-aryl skeleton) and amadandiol (aryl-C15-aryl skeleton) [56].

#### Monomeric and dimeric phenylpropene derivatives (Fig. 2)

Monomeric phenylpropene derivatives can be best represented by calebin A, 400-(4000- hydroxyphenyl-3000-methoxy)-200-oxo-300-butenyl-3-(40-hydroxyphenyl)-propenoate and 400-(4000-hydroxyphenyl)-200- oxo-300-butenyl-3-(40 -hydroxyphenyl-30 -methoxy)-propenoate which were derived from *C. oligantha* rhizome [56]. While 1-*p*-coumaroyl-cinnamic acid, 1-feruloyloxy-2-methoxycinnamic acid and 1-feruloyloxy-cinnamic acid isolated from *C. chuanyujin* rhizome were belonging to the dimeric polypropene derivatives [57].

#### Alkaloids (Fig. 2)

The *Curcuma* alkaloids were mainly isolated from the root extracts of different species where aurantiamide and curcuminol I were defined from *C. wenyujin* (root) [58, 59] and 2-(20 -methyl-10 -propenyl)-4,6-dimethyl-7-hydroxyquinoline was reported from *C. longa* [60].

#### Flavonoid glycosides (Fig. 2)

Different classes of flavonoids and their derivatives were isolated from genus *Curcuma*. Quercetin, naringenin and malvidin 3-rutinoside were reported from *C. comosa* (aerial parts), *C. longa* (rhizome), *C. zedoaria* (rhizome), *C. alismatifolia* (flower) extracts [61]. The aerial parts extract of *C. longa* showed the presence of quercetin 3-(2-galloyl-rhamnosyl-robinobioside), quercetin 3-rutinoside, quercetin 3-rhamnosyl-(1 → 2)-rhamnoside and quercetin-3-*O*-rhamnoside [62].

#### Chromone derivatives (Fig. 2)

Chromones from genus *Curcuma* were mainly represented by 2-decyl-5-hydroxy-4H-chromen-4-one, 7-butoxy4-methyl-3-pentyl-2H-chromen-2-one, 3-butyl7-hydroxy-4-methyl-6-pentyl-2H-chromen-2-one, 3-isobutyl-4-methyl-7-pentyloxy-2H-chromate2-one, 1-(4-isopropyl-2,2-dimethyl-7-propoxy-2H-(chromate-6-il)-etanon from *C. aeruginosa* rhizome [63]. Moreover, another chromone named ermanin was reported from *C. zedoaria* [39].

### Terpenes (Fig. 2)

#### Sesquiterpenes

A type of monocyclic sesquiterpene named turmerone (II) was reported from the essential oil *C. longa* rhizome [64–67]. Moreover, from *C. phaeocaulis* rhizome methanol extract a number of eudesmane sesquiterpenes were isolated namely; phaeusmanes A–H [68]. Curcumenone (cyclopropane-sesquiterpene) together with curcumanolide A and curcumanolide B were reported from *C. zedoaria* dried rhizome [69]. Two sesquiterpenoids, cadinane-type, namely; curcujinone A and curcujinone B were isolated from the ethanol extract of the root tubers of *C. wenyujin* [65].

#### Sesterpenes

Curcusesterterpene A, curcusesterterpene B and curcusesterterpene C from *C. aromatica* rhizome [56].

#### Diterpenes

Labadane diterpene such as 5S,9S,10S,15R-(-)-curcuminol D and 5S,9S,10S,15R-(-)-curcuminol H was reported from *C. kwangsiensis* [56] while the rhizome extract of *C. wenyujin* contained mainly isopimarane-type diterpenes named curcumrinol A and curcumrinol B [56].

#### Triterpenes

Three triterpenes were isolated from the rhizome of *C. longa* namely; hop-17(21)-en-3b-ol, hop-17(21)- en-3b-yl acetate and hopenone I [56]. Different triterpenoids were identified from the rhizome of *C. zanthorrhiza* through UPLC/MS. The identified compounds were zedoarolide B, zedoalactone B, curcumenolactone C, bisacurone, zedoarol, camphor, 13-hydroxygermacrone, *iso*-velleral, coronarin E, curcumenolactone A, *gamma*-bicyclohomo farnesal, curcumalactone, agaruspirol, spathulenol, zedoaraldehyde, gweicurculactone, arturmerone, curzerenone, curzerenone, curcumene and xanthorrhizol [70].

*Curcuma mangga* Val. rhizome *n*-hexane extract showed the presence of zedoarondiol, curcumenol, curcumenone and 13-hydroxygermacrone [71]. Similarly, difurocumenone, aerugidiol, zedoalactone A, zedoalactone B, zedoarondiol, curzerenone, furanodienone, furanogermenone, zedoarol, curcumenol, isocurcumenol, aeruginolactone, aeruginone, furanodiene, germacrone, zederone, dehydrocurdione, dehydrocurdione, aeruginon, curcumenon, pyrocurzerenone, dehydrochromolaenin, curzeone, linderazulene, 8,12-epoxy-1(10),4(15),7,11-germacratetraen-6-one, 1(10),4(5)-diepoxygermacrone, isoaromadendrene epoxide were reported from *C. aeruginosa* rhizome [26].

Temu mangga (*Curcuma manga*) rhizomes from Indonesia was extracted with *n*-hexane which was then subjected to LC/MS. Triterpenoids were the major identified compounds such as zedoarondiol, curcumenol, curcumenone and 13-hydroxygermacrone [72]. The ethyl acetate extract of *C. kwangsiensis* was rich in (12*Z*,14*R*)-7*β*-hydroxylabda-8(17),12-diene-14,15,16-triol, (12*Z*,14*S*)- 7*β*-hydroxlabda-8(17),12-diene-14,15,16-triol and (4*S*)-hydroxy-(8)-methoxy-(5*S*)-(H)-guaia1(10),7(11)-dien-12,8-olide [64]. Different triterpenoids *viz.* curcumenol, 7*α*,11*α*-epoxy-5*β*-hydroxy-9-guaiaen-8-one, curdione, (1S, 4S, 5S,10S)-germacrone, zederone, curcumanolide A, curcumanolide B, gajutsulactone B and wenyujinin C were isolated from the petroleum ether extract of the root tubers of *C. wenyujin* [65].

#### Phenolic acids (Fig. 2)

Gallic acid, protocatechuic acid, gentisic acid, vanillic acid, syringic acid, *p*-coumaric acid, caffeic acid, ferulic acid were defined from *C. amada* [73] while vanillin and (*E*)-4-(4-hydroxy-3-methoxyphenyl)but-3-en-2-one were isolated from *C. longa* [74].

#### Volatile constituents

A lot of the well-known volatile components were reported from various species of genus *Curcuma*. The identified volatile constituents belonged to both the oxygenated and non-oxygenated hydrocarbons *viz. α*-pinene, *α*-fenchene, camphene, camphene hydrate, *β*-pinene, 3-carene, 2-thujene, sabinene, 6-isopropylidenebicyclo[3.1.0]hexane, bi-cyclo [3.1.0] hexane-3-one, 1,8-cineol (eucalyptol), norbornyl acetate, *trans*-chrysanthenyl acetate, borneol, isoborneol, 4,7,7-trimethyl-bicyclo [2.2.1] heptan-1-ol, endo-1,5,6,7-tetramethylbicyclo[3.2.0]hept-6-en-3-ol, 2-oxabicyclo (3.2.1) octane 1.4-dimethyl-8-methylene, terpene-4-ol, isopulegol, *p*-mentha-1,4 (8)-diene, 2-menthen-1-ol, L-carveol, menth-1-en-9-ol, *α*-terpineol, *β*-terpineol, camphor (1*R*,4*R*), acetophenone, *p*-cymen-8-ol, thymol, 2-methoxy-4 vinylphenol, (*E,E,E*)-3,7,11,15-tetramethylhexadeca-1,3,6,10,14-pentaene, 2,6,11,15-tetramethylhexadeca-2,6,8,10,14-pentaene, *Z*-*α*-farnesene, *Z*-*β*-farnesene, myrcene, (*E*)-*β*-ocimene, (*Z*)-*β*-ocimene (cisocimene), *cis*-dihydroocimene, *trans*dihydroocimene, hexadecane, heptadecane, octadecane, 2-methylheptan-3-ol, nonan-2-ol, *n*-nonacosan-1-ol, undecanol, L*-*octen-3-ol and linalool which were identified from the essential oil or *n*-hexane extract of *C. aeruginosa* (rhizome), *C. aromatic* (leaves), *C. manga* (rhizome), *C. caesia* (leaves), *C. longa* (leaves, rhizome), *C. amada* (leaves, rhizome), *C. angustifolia* (rhizome), *C. zedoaria.*(rhizome), *C. inodora* (leaves, rhizome), *C. purpurascens* (leaves, rhizome) and *C. xanthorrhiza* (rhizome) using GC/MS analyses [18].

### Total phenolic and total flavonoid contents

*Curcuma aeruginosa* is a rhizomatous medicinal plant with beneficial pharmacological activities. The aim of this work was to analyze the agro-morphological, extract yield, and phenolic content often *C. aeruginosa* accessions which were collected from different locations in Indonesia. Cultivation was carried out in the open field in West Java of Indonesia using a completely randomized design. Qualitative and quantitative parameters were used to investigate agro-morphological traits. Total phenolic and total flavonoids contents were determined in ethanol extracts of samples. The plants were phenotypically diverse, in which there were significant variations among the ten *C. aeruginosa* accessions in number of leaves, plant height, number of shoots, fresh weight of rhizome, and dry weight of rhizome characters. Variability in the totalphenolic and total flavonoid contents ranged from 29.08–46.92mg GAE/g, and 21.31–33.81mgQE/g, respectively. Six accessions had high phenolic content and extract yield [75].

The antibiofilm and antioxidant activities associated with turmeric were the main focus of thestudy. Antibacterial activity was explored against bacteria isolated from dental plaques and dental unit water lines exhibiting resistance against antibiotics and biocides respectively. This study provides a comparison of the natural plant extract against synthetic mouthwash, chemicals andcommonly prescribed antibiotics. Methanol extract was more effective as compared to otherextracts. Minimum inhibitory concentrations (MIC) ranged from 2.5-10mg/ml. Time based killing kinetic assay showed a significant reduction of bacterial load with increasing concentration of turmeric. Micro titer plate assay indicated significant inhibition of biofilm formation in cellstreated with turmeric extract. Phytochemical screening of plant extracts showed the presence of vital secondary metabolites. Flavonoid content and total phenolic content varied among extracts, phenolic content for methanol extract was 61.669mg GAE/gm dry extract and flavonoid content was 3.119mg quercitin/gm dry extract. The values of ferric reducing power were in the range of 5.55–15.55mmol of FeSO_4_ equivalent/liter of the extract. Antioxidant activities and total phenolic content of the turmeric extracts had significant positive correlation. On the basis of these resultsturmeric may confidently be recommended as natural antibiofilm and antioxidant agent [76].

### Genus *Curcuma* and endophytes

Genus *Curcuma* carries a large variety of plant-associated fungi either as rhizosphere-associated microbial species or as endophytes. Both rhizospheric and endophytic species are directly or indirectly involved in growth promotion and disease management in plants and also play an important role in the modulation of morphological growth, secondary metabolite production, curcumin content, antioxidant properties, *etc*. [77]. The endophytes are usually defined as “non-pathogenic microorganisms that colonize the internal parts of the plant, helping in enhancing the plant growth mechanism and protection by helping the host plants to produce phytohormones like indole acetic acid, gibberellins and cytokinin which promotes the growth of the plant’. They act as a reaction elicitor that help to induce the production of certain plant secondary metabolites. The produced 2^ry^ metabolites may protect the plant from pests and insects also it carries antimicrobial properties against plant pathogens, thus protecting the host during abiotic and biotic stress conditions. Part of these produced 2^ry^ metabolites are of valuable medicinal role [78].

*Ceratobasidium ramicola* IBRLCM127 was detected as an endophytic fungus from the rhizomes of *C. mangga*. This fungal endophyte presented promising antimicrobial activity upon screening using agar plug assay where it inhibited eleven bacteria. Gram-positive bacteria were most susceptible to such endophytes (inhibition zones of ≥ 21 mm). Gram-negative bacteria, (*Proteus mirabilis*, *Yersinia enterocolitica*, *Escherichia coli* IBRL 0157, *Salmonella typhimurium*, *Klebsiella pneumoniae* ATCC 13883 and *Acinetobacter antratus*) were the most susceptible (inhibition zones ≥ 21 mm) while *Pseudomonas aeruginosa* ATCC 27844 was the least susceptible (inhibition zone 11 to ≤ 20 mm). MIC and MBC for Gram-positive bacteria were both in the range of 125.00 to 500.00 µg/mL, while for Gram-negative bacteria the range of 125.00 to 250.00 and 250.00 to 500.00 µg/mL were recorded, respectively [79].

The search for endophytes to produce L-asparaginase is gaining much attention recently. *Talaromyces pinophilus*, an endophytic fungus isolated from the rhizomes of *C. amada* was tested for its ability to produce the L-asparaginase enzyme. The enzyme production was performed *via* Submerged Fermentation (SmF) followed by Solid-state Fermentation (SSF). Optimal concentrations of various metal salts were incorporated in the optimized liquid medium, by one-factor-at-a-time experiments. To further enhance L-asparaginase production, SSF was carried out using Polyurethane Foam (PUF) as inert support impregnated with the optimized liquid medium. This study highlighted the benefits of carrying out SSF with PUF, using the same liquid medium optimized for SmF—a novel approach to enhance the enzyme yield (in our case an increase of about 27% was observed) [80]. Similarly, another study evaluated the potential purification and isolation of l-asparaginase from the same endophytic fungus mentioned above. Maximum enzyme activity could be achieved at pH 8.0 and with temperature 28 °C. The enzyme Exhibits 95% and 98% of its total activity at physiological pH and temperature, respectively. The enzyme activity is largely unhindered in the presence of metal ions such as Ca^2+^, Cu^2+^, Fe^2+^, Mg^2+^, Mn^2+^, Zn^2+^. Increase in the enzyme activity in the presence of thiol groups and reduction in the same upon addition of thiol blockers indicates the involvement of cysteine in the enzyme’s catalytic activity. The enzyme is a heterodimer of 62 kDa and 39 kDa. The enzyme has a *K*_m_ of 6.4 mM, its turnover number towards L-asparagine is 286.3 s^−1^. The enzyme has 16% glutaminase activity; its *K*_m_ towards glutamine is 13.3 mM and turnover number is 54.6 s^−1^ [81].

*Ceratobasidium ramicola* IBRLCM127 is one of the endophytic fungi isolated from *C. mangga* rhizome. This endophyte had potential anti-candidal activity. The fungal ethyl acetate extract showed significant inhibitory zones against *C. albicans* (MIC = 2.5 mg/ml [82]. *C. longa* showed the presence of forty-four endophytic. The isolated endophytes were fermented in Potato Dextrose Broth (PDB) media, filtered, extracted with ethyl acetate and then were analyzed by TLC and screened for their antioxidant activity. Six of the isolated endophytes presented antioxidant activities more than 65% namely; K.Cl.Sb.R9 (93.58%), K.Cl.Sb.A11 (81.49%), KCl.Sb.B1 (78.81%), KCl.Sb.R11 (71.67%) and K.Cl.Sb.A12 (67.76%) from Sukabumi and K.Cl.Cb.U1 (69.27%) from Cibinong [83].

Five phytoconstituents were characterized from the endophytic fungus EZG0807 associated with the root of *C. wenyujin*. The isolated compounds were namely; curcumin, cinnamic acid, 1,4-dihydroxyanthraquinone, gibberellic acid, and kaempferol [84]. An endophytic fungus, *Chaetomium globosum*, was isolated from *C. wenyujin*. This endophytic fungus was found rich in chaetoglobosin X, erogosterol, ergosterol 5*α*,8-peroside and 2-methyl-3-hydroxy indole. Chaetoglobosin X exhibited a broader antifungal spectrum and showed the strongest cytotoxic activity against H_22_ and MFC cancer cell lines [85]. The ability of *C. longa* associated endophytic fungi to convert the main metabolite curcumin was investigated. *Diaporthe* sp. (an endophytic filamentous fungus) converted curcumin into four colorless derivatives such as (3*R*,5*R*)-tetrahydrocurcumin, neohexahydrocurcumin, (3*S*,5*S*)-octahydrocurcumin and *meso*-octahydrocurcumin [86].

### Biological activities of genus *Curcuma*

Different biological processes had been linked to aging including oxidative stress, inflammation, neurological disorders, hyperlipidemia and cancer. *Curcuma* species extracts were reported to combat such changes as detailed below in Table 1.

**Table 1:** Antiaging biological activities of genus *Curcuma*

*Curcuma* species	Common name	Biological activity	Experiment/test	Dosage/concentration	Main findings/results	Reference(s)
*C. caesia*	Black turmeric	Antioxidant	DPPH *α*-amylase inhibition	18μg/mL for the DPPH assay and 26μg/mL for the *α*-amylase inhibition assay	DPPH (IC_50_ = 299.18μg/mL) *α*-amylase inhibitory (IC_50_ = 50.16μg/mL)	[88]
			DPPH	48 μg/mL	(IC_50_ = 1.487μg/mL)	[89]
*C. aromatica*	Wild turmeric		DPPH, lipid peroxidation assay and protein denaturation inhibition	50 µl extract	DPPH (IC_50_ = 0.55 ± 0.02mg/mL) Lipid peroxidation (IC_50_ = 0.60 ± 0.10mg/mL). Protein denaturation inhibition (65.97 ± 4.68%)	[90]
*C. xanthorrhiza*	Javanese turmeric		DPPH	1ml of extract	(IC_50_ = 0.177–0.615mg/mL)	[91]
			DPPH, CUPRAC (Cupric ion Reducing Antioxidant Capacity), lipase enzyme inhibition and BCB (*β*-Carotene Bleaching) methods	–	DPPH (IC_50_ = 255.36 ± 1.87 µg/mL), CUPRAC (IC_50_ = 150.42 ± 13.71 µmol QE quercetin/100 g), and Lipase inhibition (115.79 ± 3.44 µg/mL) and BCB (IC_50_ = 232.15 ± 1.99 µg/mL)	[92]
*C. longa* (*C. domestica*)	**––-**		Reducing power assay and the cyclic voltammetry technique	2 mL extract extract, 2.50 mL of phosphate buffer (0.20 M, pH = 6.60) was added followed by addition of 2.50 mL of 1% K_3_Fe(CN)_6_	Reducing power = 45.632 ± 2.026	[93]
*C. longa* [variety: Ryudai gold (RD) and Okinawa ukon], *C. xanthorrhiza*, *C. aromatica*, *C. amada* and *C. zedoaria*	**––-**		DPPH, ORAC and 2-deoxyribose(2-DR) oxidation assay	80 μL samples at different concentrations (10, 25 and 50 μg/mL)	DPPH (IC_50_ = 26.4μg/mL), ORAC (14,090μmol Trolox equivalent/g extract), reducing power absorbance (0.33) and hydroxyl radical scavenging activity (IC_50_ = 7.4μg/mL)	[94]
*C. longa*	**––-**	Neuroprotective	Cholinesterase and glycogen synthasekinase-3*β* inhibition	1 mg/mL extract reached 4.2 μg/mL after dilution with buffer	Weak to moderate acetylcholinesterase and butyryl cholinesterase inhibition	[95]
*C. amada*	Mango ginger		Brain of F_1_ progeny of Danio rerio (Zebrafish) which has been gestationally exposed to the neurotoxicant nickel chloride	150µg	↓Anxiety behavior in the F_1_ progeny of exposed fish co-treated *C. amada* Marked improvement in the memory and learning pattern in *C. amada*-treated fishes	[96]
Curcumin	**––-**		Diabetic rats’ brains	–	↓Malondialdehyde level in the brain of diabetic rats. Protective effects against glutamate neurotoxicity in the male albino rats Corrected cognitive impairment ↓Apoptosis in the cerebral cortex	[10]
*C. aromatica*	Wild turmeric	Anti-inflammatory	Effect on inflammatory mediators	200 μg/mL extract	Inhibit in NF‐κB activity (IC_50_ = 46.9 ± 8.6)	[97]
*C. longa*	**––-**		Albumin denaturation, proteinase inhibitory activity, membrane stabilization and anti-lipoxygenase activity	0.25 mL extract in 2 M borate buffer (pH 9.0)	Significantly ↓ albumin denaturation and proteinase activity Stabilized RBCs membrane from haemolysis ↓Lipoxygenase activity	[98]
*C. singularis* Gagnep	**––-**	Cytotoxic and anti-proliferative	In vitro cytotoxicity against different gastric cancer cell lines	50 μg/mL	Cytotoxicity was concentration-dependent. ↑Bax/Bcl-2 ratio ↑ release of cytochrome C Activation of caspase-3/-7, caspase-9 and cleavage	[99]
*C. longa* (Curcumin in tiny nano-molecules)	**––-**		mTOR in breast cancer growth	–	Curcumin can act as an anti-cancer drug especially when using lipid-coated nano-particles for better drug delivery	[100, 101]
*C. longa* (Curcumin-quercetin in lipid carrier)	**––-**		Oral squamous cell carcinoma	–	↓AKT/PI3K signaling pathway ↓Cyclin A/D/E expression Helps G1/G2/M phase arrest Nanostructured lipid-carriers improved loading capacity of the drug combination and improve site-specific targeting	[102]
*C. aeruginosa* Roxb	temu hitam	Cytotoxic and anti-proliferative	A-549 human lung adenocarcinoma and HeLa cell lines	5, 10, 20, 40, 80 & 100 µg/ml extract	↑Caspase 3 and 8 expressions Apoptosis in cancer cells	[66]
*C. longa* and *C. aromatica*	**––-**		Human cervical cancer cell line (MTT assay)	6.25, 12.5, 25, 50 & 100uL/mL extract	Cytotoxic activity (IC_50_** = **6.25-100ul)	[103]
*C. caesia* Roxb	Black turmeric		2,3,5-Triphenyltetrazolium chloride (TTC), 2',7'-dichlorofluorescein diacetate (DCFDAH 2) assays and in vitro DNA protection assay	2.5, 5 and 10 µg/ml extract	↓Oxidative DNA damage in vitro	[104]
*C. amada*	Mango ginger		Immuno-cyto-chemical analysis (expression of apoptosis-associated proteins Bax, Bcl-2, and p53)	–	↑Pro-apoptotic proteins p53and Bax expression in cancer cells ↓Anti-apoptotic protein Bcl-2 expression	[105]
*C. longa*	––-	Anti-hyperlipidemic	Hypercholesterolaemic male albino rats	200mg/kg of the extract orally to rats	The* Curcuma* extract caused hypolipidemic effect in tested rats.	[106]
*C. zedoaria* Roscoe	White turmeric or zedoary		Clinical study on human subjects	–	↓Serum total cholesterol after 60 days Significant increase in HDL-cholesterol after two months. Pronounced decrease in serum LDL-cholesterol and triglycerides.	[105]

*Curcuma* or turmeric is famous by its potent phytochemicals namely curcumin, demethoxycurcumin, bis-demethoxycurcumin, zingiberene, curcumenol, curcumol, eugenol, tetrahydrocurcumin, triethylcurcumin, turmerin, turmerones, and turmeronols. Although curcumin is the most abundant and well-known active ingredient of curcuma, many research articles had addressed the idea of the presence of other active components synergizing with curcumin. Three main curcumin analogs (diferuloylmethane, demethoxycurcumin and bis-demothycurcumin) were causing such synergism. In some cases, bisdemethoxycurcumin was found more potent compared to curcumin and in other cases a mixture of the two compounds lead to higher activity. On the other hand, tetrahydrocurcumin (a metabolite of curcumin) has been shown to be active in some systems and not in others. Despite extensive research on *Curcuma* and its active metabolites, a lot of questions are still to be answered including the optimum dosage for effective biological activities, bioavailability and serum levels of curcumin as the main active metabolite is low also the concentration of curcumin in tissues and in cell culture [87].

### Toxicity studies on genus *Curcma*

A study was conducted to evaluate the possible toxic effects of two hundred compounds from turmeric in regard of bacterial mutagenicity, rodent carcinogenicity and human hepatotoxicity. One hundred and eighty four compounds were found toxic while one hundred and thirty six were mutagenic, one hundred fifty three compounds were carcinogenic and sixty four were hepatotoxic [107]. According to both the FAO/WHO reports on food additives, the daily intake of curcumin should not exceed 0–1mg/kg body weight for no adverse effects. Chronic toxicity studies on curcumin concluded that it can be administered safely at oral doses of up to 8 g per day. Moreover, in vivo studies in rats found that the oral administration of 1 g/kg of curcumin resulted in that it was excreted unmodified with faeces (about 75%) with minor appearance in urine. It is worthy noted also that, curcumin showed extremely low serum levels. The average peak serum concentrations after taking 4mg, 6mg and 8mg/day of curcumin were 0.51 ± 0.11µM, 0.63 ± 0.06µM and 1.77 ± 1.87µM, respectively [108].

### Bioavailability and metabolic pathways involved in* Curcuma* consumption

Clinical trials on curcumin human consumption had concluded that the compound is safe up to 12 g/day however it suffers from poor bioavailability. This low bioavailability can be attributed to its poor absorption, rapid metabolism, and rapid systemic elimination. Many research articles were carried out in order to improve curcumin bioavailability through many approaches including: the use of adjuvant like piperine that interferes with glucuronidation, incorporating the compound in liposomes, curcumin nanoparticles, developing curcumin-phospholipid complex and structural modification of curcumin into more bioavailable leads. Curcumin and its derivatives has een proven as potent antioxidant, anti-inflammatory and anticancer agents thus improving their bioavailability as a future research target may lead to the development of promising drug leads and nutraceutical formulations that are potent against many age-related diseases and disorders [109–111].

## Discussion

Natural products show diverse chemical classes accounting for more than 200,000 of functional metabolites belonging to different classes such as flavonoids, phenolic acids, phenylpropanoids, terpenoids and alkaloids [112].

Aromatic and medicinal plants serve as abundant sources of natural products, many of which possess significant benefits for human health. These benefits include as the prevention and/or treatment of various cancers, as well as inflammatory, cardiovascular and neurodegenerative diseases. Apart from species and/or organ specific accumulation patterns, natural products occur in response to various abiotic and biotic stresses. They serve diverse biological functions, acting as attractants, repellants and other olfactory signaling molecules involved in olfactory communication. Additionally, they play an essential role in growth and development [113].

Genus *Curcuma* has long been tied to the idea of age reversal through its vital ingredient curcumin which belongs to a wider umbrella named the curcuminoides which comprises the diphenylpentanoids, diphenyheptanoids, diarylheptanoids, *etc*. Curcumin showed its significant and powerful antiaging activity through many mechanisms *viz.* reducing the levels of the oxidative radicals and enzymes (malonaldehyde, lipid peroxidase) in the brain tissue, reducing cell aging and increasing cell viability, repairing damaged cardiovascular endothelium, reducing artery stiffness and enhancing its elasticity and also by having a protective role against radiation damage [114–116].

Other research articles and review articles discussed either genus *Curcuma* phytoconstituents or studied the possible biological activities of curcumin as the main phytoconstituent of *Curcuma* as follows. One review article discussed the ethnobotanical properties, phytochemical constituents and biological activities of two *Curcuma* species namely; *C. longa* and *C. zedoaria* [18]. Another review article listed the main phytoconstituents and biological activities of genus *Curcuma* [56]. Moreover, Curcumin effect on the brain age-related disorders was the focus of one article where the neuroprotective, antioxidant and anti-inflammatory activity effects of curcumin were detailed. Curcumin showed neurologic protection *via* its effect on oxidative stress proteins and its anti-inflammatory activity against inflammation of the microgalia [36]. It is worthy noted here that this current review article is considered the first linking the different biological effects of the whole members of genus *Curcuma* to its possible antiaging effects through the last twenty years.

Although Curcumin and other curcuminoides represent the corner stone for the antiaging role of genus *Curcuma*, the presence of other phytoconstituents was found to be essential for such activity. Triterpenoids occurring together with curcuminodes in the *Curcuma* extract were found to play a synergestic role *via* enhancing the curcuminoides absorption due to the lipophilic nature of the terpenoids. *Curcuma *triterpenoids may also act as anti-inflammatory agents helping in the down regulation of nuclear factor KB thus reducing the oxidative stress level and modulating the aging process [117].

Curcumin despite being the main active agent of *Curcuma* with reported evidence on its efficacy and safety, it has not yet been approved as a therapeutic agent due to its low bioavailability, instability at physiological pH, insolubility in water, slow uptake by cells and rapid metabolism inside the cells [108, 118].

Alzheimer, Parkinson’s and dementia together with physical health deterioration are normal players in the process of aging leading to many limitations on people quality of life. The presence of strong antioxidant, cytotoxic and anti-inflammatory agents such as those reported from genus *Curcuma* may help in the prophylactic and treatment measures related to aging [119].

## Conclusions and future perspectives

In conclusion, the genus *Curcuma,* with almost eighty species, carry a lot of interesting phytoconstituents belonging to many chemical classes. The rhizomes followed by the roots represent the most studied part of genus *Curcuma* being rich in the main phytoconstituents. The vast array of biological activities played by the members of genus *Curcuma* has positioned them as an important part of the research targets, especially those against aging and senile-related diseases. Alzheimer, Parkinson’s and dementia together with many neurologic defects that accompany aging are usually the result of oxidative stress and prolonged inflammation thus the presence of strong antioxidants and anti-inflammatory effects played by *Curcuma* extracts may help in slowing down or reversing such deteriorations. More research is needed concerning the other parts of the genus *Curcuma* such as the leaves, seeds, and stems. The biological activities are in most cases due to the high curcumin content although few research articles addressed the synergistic role that may be played by the other ingredients of the *Curcuma* extracts. Moreover, molecular mechanisms confirming the reported biological activities may be a noteworthy future research interest.

## Data Availability

Not applicable.
